# Report of Eighteen Cases of Typhoid Fever

**Published:** 1893-01

**Authors:** H. A. West

**Affiliations:** Galveston


### DANIEL'S TEXAS MEDICAL JOURNAL.

ESTABLISHED JULY, 1885.

PUBLISHED MONTHLY.—SUBSCRIPTION $2.00 A YEAR.

Vol. VIII. AUSTIN, JANUARY, 1893. No. 7.

### Original Contributions.

For Daniel's Texas Medical Journal.

### REPORT OF EIGHTEEN CASES OF TYPHOID FEVER.

BY H. A. WEST, M. D., GALVESTON.

[Read before the Galveston County Medical Society,* at Galveston, November 21, 1892.]

MR. PRESIDENT AND FELLOW MEMBERS:—Dr. Smith expected to have been present this evening, to demonstrate the lesions of typhoid fever in two specimens obtained from patients of mine, who have recently died from intestinal perforation. I will leave it to him to show you these specimens, and to discuss the pathology of typhoid fever, at a future special meeting of the Society. Preliminary to this demonstration, however, I will give you brief clinical details of these two cases which proved fatal, as well as of sixteen others now under treatment in the Sealy Hospital, or lately discharged therefrom.

This specimen was obtained from J. P., aged 36, native of England, an assistant engineer in a British steamer. He entered the hospital October 15, Saturday morning, giving the following history: He had been complaining for about three weeks, with indefinite symptoms, loss of appetite, slight fever and chilly sensations, but did not consider himself sick enough to quit work

*By unanimous vote of the Society, DANIEL'S TEXAS MEDICAL JOURNAL was made the official organ of the Galveston County Medical Society, December 20, 1892.

until the day preceding his admission into the hospital. He was then taken with sudden abdominal pain, high fever, and vomiting. When I saw him, the temperature was 103° F.; pulse, 120, small and thready; abdomen swollen largely, tympanitic, and tender, especially about the umbilicus; bowels were constipated, an injection of sulphate of magnesium failing to bring away any fœcal matters. Recognizing an acute peritonitis, I called in Dr. Thompson, to consider the question of an exploratory laparotomy. It was decided that such an operation offered the only chance to save the patient's life. It was accordingly done the next day. The small intestines were found to be matted together by recent adhesions, intensely congested, and distended by gaseous and fluid contents. There was quite a collection of liquid fœcal matters, mixed with pus and fibrinous exudations, in the abdominal cavity, with a partial encapsulation in the right iliac region. It was found to be impossible to ascertain the cause of this condition, without relieving the distended part. This was done with a trocar, and about two quarts of thin, light-colored fluid, containing fœcal matter, was evacuated. In following up the coils of intestine, the cause of the trouble was found in a perforation, round, and about the size of the finger nail. Both openings were carefully sewed up with Lambert's sutures, and the abdomen thoroughly washed out, and closed. The patient rallied from the shock of the operation, but, as was expected, died that very night. The autopsy revealed the characteristic lesions of enteric fever. In addition to the ulcer which had caused perforation, there was extensive ulceration involving Peyer's patches and the solitary follicles in the lower portion of the ilium. The spleen and mesentary glands were enlarged.

The second specimen is from a patient giving a somewhat similar history. M. D., aged 34, native of France, was admitted on October 19, stating that he had been sick about twelve days, his illness having begun in Houston, where he stayed five or six days before coming to this city. He was not confined during this time, having no place to stay, and suffered from exposure, improper and insufficient food, as well as other hardships. Upon admission, symptoms of enteric fever were well marked. The tongue was red, fissured and dry; there were anorexia, constipation, some tympanites, and severe pain in the right iliac region. The spleen was enlarged, and rose-colored spots were found upon the abdomen, the temperature ranging from 101° F. in the morning to $104\frac{4}{5}$° F. in the evening. The acute iliac pain subsided gradually. The patient was ordered a milk diet, and the bowels to be relieved by enemata. Turpentine stupes were applied to the abdomen, and a mild opiate and expectorant mixture given to relieve a troublesome cough. The patient got along pretty well until the night of October 28, when he was taken with terrible pain in the right iliac region; the temperature suddenly fell from 104½° to 98½° F. There was every symptom of collapse from intestinal perforation; small, thready pulse, cold sweating, uncontrollable vomiting, death taking place October 30. Autopsy showed intense peritonitis, extensive ulceration of Peyer's patches and solitary glands; enlargement of the spleen, enlargement and induration of the mesenteric glands. A few inches above the cæcum, one patch had sloughed, perforating the ilium by an opening as large as the end of the thumb.

Here, then, are the only fatal cases out of the series, and both ambulatory; one actually keeping at his work until the deadly ulcer had eaten a hole in his gut; the other knocking about from pillar to post, with extensive ulceration, and several ulcers on the point of perforation. Comment is unnecessary. The lives of these patients, I think, might have been saved had they been brought under observation earlier, and placed upon proper treatment. Another prominent fact illustrated is, that patients with typhoid fever may actually reach the day of their death without presenting any symptoms whatever to ordinary observation, and not knowing themselves that they are seriously sick.

Now, as to the other sixteen cases. I have brief histories of them here, but will not weary you by going into details. It will be sufficient to analyze them as regards the origin, symptomatology and results.

Any one might suppose that an epidemic of typhoid fever was prevalent in Galveston, judging by the number of such cases which have crowded my wards in the Sealy Hospital since the first of October; but as will be shown, two-thirds of the number were from elsewhere. The first point worthy of notice is, that all are males. There has not been a female patient with typhoid fever in my service, up to the present time, this season. They come from widely different parts of the country, as follows: From Rouen, France, one seaman; from Tampico, Mexico, two seamen; from Liverpool, England, three; from various points in the interior of Texas, seven. Of these latter, one was from Wharton county, one from San Antonio, one from Fort Worth, one from Rock Island, North Texas, and three were traveling through the State. Five of these patients belonged to Galveston. Of all of these eighteen cases, twelve had diarrhœa, and the other six suffered from constipation during the entire course of the disease. The fever, in nearly all of them, came on gradually, accompanied with headache, anorexia, and general malaise. The tongue presented a very similar appearance in all, slightly coated at first, red at the tip and edges, and, later, getting dry, glazed and fissured. All, without exception, had the rose-colored spots; none had any pulmonary complications, and only three had severe nervous symptoms, which fact, no doubt, can be explained by the prophylactic treatment, that is, careful attention to cleansing of the mouth and cold water baths. Epistaxis occurred in one case; hemorrhage from the bowels in none. Two, so far, have had relapses. Two, as mentioned already, died from intestinal perforation. The diagnosis has presented no difficulty in any of these cases. Most any tyro could have gone through the wards and differentiated the cases of typhoid fever from those of malaria.

Another fact of interest is, that in one instance in which there was positive evidence of the existence of both typhoid and malarial infections, there was no commingling of the two in one person. For example, an English tramp steamer, which had been detained thirty days at Tampico, has furnished a number of both forms of fever. The malarial form was well marked, and invariably characterized by the presence of the parasites in the blood, and rapidly yielded to quinine, while the typhoid cases showed no evidence whatever of malarial complication. Not one of these cases, by any stretch of the diagnostic imagination, could be called typho-malarial. This confirms my previous observation, that the two forms of fever are usually easy to differentiate, especially with the careful use of the thermometer and microscopical examination of the blood. I have had, in my wards, a number of patients with slow forms of continued malarial fevers, but such fevers bear no resemblance to typhoid.

Upon the subject of treatment, I have a few words to say. Dr. Osler never made a truer statement than when he said that "it had taken the medical profession a long time to find out that typhoid fever is not a disease to be treated by medicines." This is only another way of saying that careful nursing and feeding will accomplish more than drugs. There is no disease in which a careful and conscientious nurse can accomplish more than in typhoid fever. It is her business, under the direction of the physician, to carefully attend to all of those important details which may be included under the terms cleanliness and antipepsis, and which not only add to the peculiar comfort of the patient, but also improve his chances for recovery by preventing serious complications. She should know the kind of diet suitable for such a patient, and how to prepare and administer it. In my experience, the proper feeding of a patient with typhoid fever, is the most difficult part of the treatment. It is even more troublesome to restrain the patient's appetite in the period of convalescence, than it is to get him to take sufficient nourishment during the height of the fever. Milk is the acknowledged ideal food. Its administration should be carefully watched, and digestion aided by admixture with lime-water, some of the alkaline carbonated waters, or it should be given after previous fermentation, in the shape of "matzoon," or "kumyss," etc. By the use of milk in this way, the scanty gastric juice is not all consumed in the coagulation of the casein, and some is left for its conversion into peptones. Buttermilk, for a similar reason, is advisable. Cream and whey, or a little ice cream, may also be used.

While I believe that milk is by far the best food, patients are apt to get tired and disgusted with it, hence the necessity of adding to the dietary some carefully prepared animal broths, or some light farinaceous food, such as arrow-root or sago, and of assisting in the digestion of all by the administration of pepsin. As regards alcohol, I do not believe that there exists any necessity for its routine administration. When there is an indication for stimulants as evidenced by a failing circulation and an exaggeration of the nervous phenomena, then alcohol and strychnine should be given with a free hand, but the emergency over, they should be discontinued. Next in value as a therapeutic measure, in my opinion, is the cold bath, introduced nearly a century ago, by Cumi. This practice had fallen into disuse until revived by Brand. I shall not enter here into details as to the modus operandi and management of the baths, but will close by giving this treatment my emphatic endorsement. In my hands, it has proven of infinite value.

